# Chemical characterization of hibiscus rosa-sinensis plant fibers facilitated through design of experiments and artificial neural network hybrid approach

**DOI:** 10.1038/s41598-024-73503-8

**Published:** 2024-09-28

**Authors:** J. P. Supriya, Raviraj Shetty, Nithesh Naik, Srinivasulu Maddasani, Adithya Hegde

**Affiliations:** 1 Department of Mechanical and Industrial Engineering, Manipal Institute of Technology, Manipal Academy of Higher Education, Manipal, 576104 India; 2 Department of Chemistry, Manipal Institute of Technology, Manipal Academy of Higher Education, Manipal, 576104 India

**Keywords:** Chemical Treatment, Hibiscus Rosa-Sinensis, Sodium Hydroxide, Acetic Acid, Potassium permanganate, Crystallinity Index, Thermo-Gravimetric analysis, Engineering, Materials science

## Abstract

The integration of natural fibers into Fiber Reinforced Polymers (FRPs) has emerged as a promising avenue for sustainable and high-performance composite materials. Natural fibers, derived from plants, offer notable advantages such as renewability, low cost, and environmental friendliness. Among these natural fibers, Hibiscus Rosa-Sinensis (HRS) plant fibers have gained significant attention owing to their widespread availability and unique mechanical properties. In this study, HRS fibers were chemically treated using Sodium Hydroxide (NaOH), Potassium Permanganate (KMnO_4_), and Acetic Acid (CH_3_COOH) at different weight percentages (3, 4, 5 Wt.%) and solutionizing times (1, 2, 3 h) based on Taguchi’s L_27_ orthogonal array. The fibers, extracted from epidermis of the stems, underwent cleaning and chemical treatment after water retting. The crystallinity index, determined via X-ray Diffraction (XRD), indicated a maximum value of 65.77%. Thermo-gravimetric analysis (TGA) exhibited a degradation temperature of 365.24 °C and a material loss of 63.11%. Potassium Permanganate treatment at 4 Wt.% and 3 h of solutionizing time has yielded the best results. Multi-Layer Perceptron Artificial Neural Network (MLP-ANN) has been successfully applied to accurately predict the output physical characteristics of chemically treated HRS fibers using experimental data. The results are in close alignment with the literature. Scanning Electron Microscopy (SEM) and Energy Dispersive X-ray Spectroscopy (EDS) analyses have provided valuable insights into the microstructure and constituents of the chemically treated HRS fibers. This research emphasises on the effectiveness of the chemical treatment process in enhancing the properties of HRS plant fibers for potential composite applications.

## Introduction

 For the last few decades, bio-composites have gained increasing significance, primarily driven by growing environmental concerns. Plant fibers are increasingly being employed as reinforcing materials in fiber-reinforced plastics, finding diverse applications in products like roofing sheets, seating, doors panels, automobiles, bullet proof, helmets, and more. This shift towards bio-composites reflects the industry’s commitment to sustainable and eco-friendly solutions^1–3^. To promote the fabrication of composites, a deep understanding of properties such as the chemical composition and bonding characteristics of the fibers is necessary. Numerous research studies have explored various techniques aimed at improving the thermal, physical and mechanical characteristics of composite materials derived from plant fibers^4^. One of the easy methods is employing chemical treatments to decrease the levels of components in plant cell wall, and impurities in plant fibers, resulting enhancements in both thermal and mechanical property. Fiber surface modification can be accomplished through chemical processes, resulting in improved adhesion between the fibers and the matrix material^5,6^. These chemical treatments typically involve the reduction of impurities and naturally occurring fluid components, such as lignin, and waxes from the surface of the fiber^7,8^. Chemical processes, including the application of sodium hydroxide, acetic acid and potassium permanganate, have proven effective in augmenting the thermal stability and mechanical characteristics of natural fibers, with the most favorable outcomes observed in composites using different plant fibers^9–11^. To explore the thermal stability of plant fiber, employed with Thermo-gravimetric Analysis (TGA) technique, which enables to systematically analyses and characterize these fibers^11,12^.X Ray Diffraction is a technique used to study crystallinity index of natural fibers and low crystallinity index value shows particles not distributed in a proper way^13,14^. SEM and EDS become essential tools for exploring the details of fibers, providing information about their elemental composition, surface properties, and structural features^15–17^. Significant knowledge regarding the hydrophilic nature of plant fibers subjected to different chemical treatments was provided by SEM measurement^18,19^. SEM stands as a high-resolution imaging technique capable of visualizing the nanoscale topographical features of materials^20^. The fibers treated with potassium permanganate exhibit enhanced crystallinity index, lower density and higher cellulose percentage lead to a promising composite with high strength and light weight^21^. The chemical treatment with sodium hydroxide results in an improved surface topography of the fiber. Excellent lignin decomposition and improved thermal stability result from the combined NaOH silane treatment^22^. The natural fiber treated with NaOH exhibited the highest density because of the contraction of fibril. The treatment with potassium permanganates reduced the water absorption capacity of the fibers. The untreated fibers enhance tensile strength compared to chemically treated fibers^23,24^. Study on synthesis of microcrystalline cellulose from Citrus x sinensis sweet orange peel fruit waste-based biomass has resulted in an alternative source of cellulose for potential value-added industrial applications^25^. Similarly studies have been conducted to find out novel materials suitable for reinforcement with polymers to form composite materials. One such effort suggests the Ficus retusa L. aerial root fiber as a potential alternative to synthetic fibers popularly used as reinforcement^26^. Different washing methods have gained popularity in enhancing the properties of natural fibers before considering them as reinforcements. Sodium Lauryl sulphate method is one popular method which has been used to process Ramie Fibers as surfactant to enhance its mechanical properties, which make them an excellent choice as reinforcement materials^27^. Recent research has been paying more attention to lignocellulosic fibers as an eco-friendly alternative to synthetic fibers in polymer composites. Among these, fibers derived from agro-waste, like the Asparagus Bean stem (ABS), show great promise. After being treated with alkali, these fibers not only increased in cellulose content but also exhibited better thermal stability and improved bonding with the polymer matrix. This treatment significantly enhanced the mechanical properties of the resulting composites, particularly in terms of flexural strength and modulus, making ABS fibers a strong candidate for reinforcing lightweight structures^33^. In the world of additive manufacturing, particularly 3D printing, using natural fibers to enhance material properties is gaining traction. Trichosanthes Cucumerina stem fibers, sourced from plant waste, have been tested in PLA filaments with encouraging results. After treating these fibers with silane, they were better distributed in the PLA matrix, and the filaments with a 6% fiber content achieved a tensile strength of 63.5 MPa. Moreover, these treated fibers contributed to more consistent filament production by reducing diameter variations. This suggests that Trichosanthes Cucumerina fibers could be a viable option for creating biodegradable components in 3D printing^34^. Another natural fiber that’s been explored is the bark from the Ficus Macrocarpa tree. When treated with alkali, these fibers showed a notable increase in cellulose content and improved thermal stability, maintaining their structure up to 378.87 °C. The treatment also resulted in a smoother surface and reduced the fiber diameter, making them more suitable for use in composites. These improvements highlight the potential of Ficus Macrocarpa bark fibers as a sustainable, environmentally friendly alternative to synthetic fibers, offering both enhanced physical properties and a smaller environmental footprint^35^.

This research paper focuses on how chemical treatments such as Sodium Hydroxide (NaOH) treatment, Potassium Permanganate (KMnO_4_) treatment, and Acetic Acid (CH_3_COOH) treatment can improve the chemical composition and physical characteristics of HRS fibers. This involves evaluating the effectiveness of each treatment in reducing unwanted components like hemicellulose and lignin while preserving or enhancing cellulose content, thus making the fibers more suitable for composite applications. To achieve this, the study employs Taguchi’s L_27_ orthogonal array design to systematically vary treatment conditions, identifying the optimal combination of chemical concentration and treatment duration. This experimental design is complemented using an Artificial Neural Network (ANN) model, which predicts the properties of treated fibers based on the experimental data. Such an approach allows for precise optimization and provides a robust framework for understanding the effects of different treatments on fiber properties. The treated fibers are thoroughly characterized using techniques such as X-ray Diffraction (XRD) for crystallinity, Thermo-gravimetric Analysis (TGA) for thermal stability, and Scanning Electron Microscopy (SEM) for surface morphology. Overall, this research not only advances the scientific understanding of natural fiber treatments but also provides practical insights that could lead to the development of high-performance, sustainable composite materials. It lays a solid foundation for future studies and industrial applications, emphasizing the role of innovative treatment methods in analysing the potential of natural fibers for modern material science.

## Methodology

### Extraction and chemical treatment of HRS fibers

In this comprehensive study, the effect of chemical treatment on physical properties of Hibiscus Rosa-Sinensis (HRS) plant fibers is meticulously investigated. HRS fibers are sourced from the dried HRS branches discarded from the Botanical Garden of Manipal Institute of Technology, Manipal, India. The fibers which are extracted from the epidermis of stems measuring 50–60 mm in length, undergo water retting process to ensure purity. These extracted fibers are cleaned to eliminate impurities and dissolved elements and dried at room temperature. Subsequently, the chemical treatment process involves the utilization of sodium hydroxide (NaOH), potassium permanganate (KMnO_4_), and acetic acid (CH_3_COOH) at varying weight percentages of 3, 4, and 5 Wt.% and solutionizing times of 1, 2, and 3 h. NaOH, a strong alkali, initiates the deacetylation process, breaking down acetyl groups and enhancing the hydrophilicity of the fibers. KMnO_4_, a potent oxidizing agent, aids in lignin degradation, effectively enhancing the fiber surface area for improved composite adhesion. Acetic acid, on the other hand, acts as a catalyst, facilitating controlled reactions and ensuring the preservation of fiber integrity. After the chemical treatment, the fibers are dried in a hot air oven, preserving the induced chemical modifications.

## Testing of the chemically treated HRS fibers

**Fig. 1:**
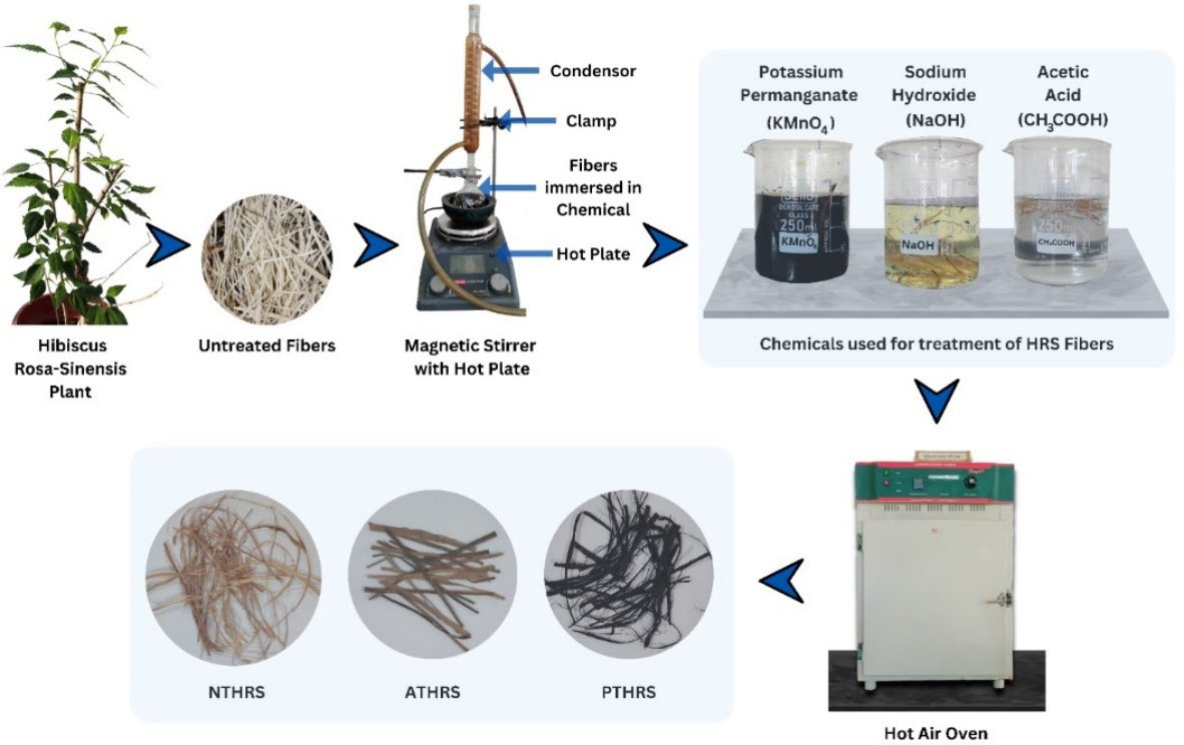
HRS fiber chemical treatment process flow diagram.

The treated fibers are further subjected to various analyses, each providing unique insights into their structural and chemical evolution. To study the structural changes, X-ray diffraction (XRD) analysis is conducted to ascertain the crystallinity index. This analysis illuminates the degree of Crystallinity within the fibers, shedding light on the efficacy of the chemical treatment process. The XRD analysis of fiber was conducted using X-Ray diffractometer (Rigaku Miniflex 600). The experimental trials are executed using CuKα radiation (λ = 1.5406 nm) at 40 kV. To derive Crystallinity (%) values from X-ray diffraction (XRD) plots, the first step involves collecting XRD data for the sample of interest using a precise X-ray diffractometer. The diffraction pattern is typically recorded in the 2θ range between 5° and 80°. Once the data is obtained, the next step is data pre-processing. During this stage, the 2θ values are converted to d-spacing using Bragg’s law, which relates the diffraction angle (2θ) to the interplanar spacing (d) and the wavelength of the X-ray radiation. This conversion is crucial for further analysis. Subsequently, the XRD pattern is baseline corrected and smoothed to enhance the signal-to-noise ratio, ensuring accurate peak identification. After these pre-processing steps, the peak intensities corresponding to the Crystallinity phases in the XRD pattern are determined. The Crystallinity Index (%) values can then be calculated by comparing the areas under the peaks associated with the Crystallinity phases to the total area under all peaks in the XRD pattern, indicating the proportion of the sample that is Crystallinity. This quantitative analysis provides valuable insights into the material’s structure and composition. Simultaneously, thermo-gravimetric analysis (TGA), specifically the STA7200 Thermal Analysis Systems from Hitachi High-Tech Science, Japan is employed to determine the degradation temperature and percentage material loss. This critical information not only elucidates the thermal stability of the fibers treated but also serves as a testament to the success of the chemical treatments in eliminating impurities and enhancing structural integrity. Material Loss is calculated by calculating the difference in weight of the fibers before and after degradation using EHP 323 Ultra-Sensitive Weight measuring balance. Scanning electron microscopy (SEM) specifically the EVO MA18 model from Carl Zeiss Ltd., is used to capture intricate details of the surface morphology of the fibers. This high-resolution imaging unveils the physical transformations undergone by the fibers, offering visual evidence of the chemical treatment’s impact. Moreover, energy-dispersive X-ray spectroscopy (EDS) specifically the EVO MA18 model from Carl Zeiss Ltd., located in Cambridge, UK. is employed to meticulously analyze the elemental composition of the treated fibers. Table 1 provides the levels and factors used in the experimentation. Figure 1 presents HRS fiber chemical treatment process flow diagram.

**Table 1:** TDOE factors and levels.

Parameters	Levels		
	1	2	3
Chemical Used	Potassium Permanganate	Sodium Hydroxide	Acetic Acid
Chemical (Wt.%)	3	4	5
Solutionizing Time (Hrs.)	1	2	3

Artificial Neural Networks have recently gained a lot of attention in various fields because of the high degrees of accuracy in predicting the output parameters of a process^28^. Back Propagation Artificial Neural Network is applied to the dataset to predict the output parameters based on the values of experimentation. Steps followed in detail are provided^29,30^.

### Step 1

Data Preparation.

The dataset, encompassing input parameters (Chemical Treatment, Chemical Wt.%, Solutionizing Time(Hrs)) and output parameters (Crystallinity Index, Degradation Temperature, Material Loss), was loaded from a CSV file. Subsequently, the data was divided into input (x) and output (y) variables.

- **```python**
- **# load data from CSV file**
- **Data = np.loadtxt(‘data.csv’, delimiter=’,‘)**
- **X = data[:, 0:3] # input parameters**
- **y = data[:, 3:] # Output parameters**
- **```**

### Step 2

Data scaling.

To ensure consistent scaling and enhance the network’s performance, the input and output variables were scaled using Min-Max scaling.

- **```python**
- **# scale input and output data**
- **scaler_X = MinMaxScaler()**
- **X_scaled = scaler_X.fit_transform(X)**
- scaler_y = MinMaxScaler()
- **y_scaled = scaler_y.fit_transform(y)**
- **```**

### Step 3

Neural network architecture.

A BPANN model was constructed using the Keras library. The model included an input layer with 3 neurons, a hidden layer with 27 neurons, as specified, and an output layer with 8 neurons corresponding to the output parameters.

- **```python**
- **# build the BPANN model**
- **Model = sequential()**
- **model.add(Dense(27, input_dim = 3, activation=’relu’)) # Hidden layer with 27 neurons**
- **model.add(dense(8, activation=’linear’)) # output layer with 8 neurons**
- **```**

### Step 4

Model compilation.

The model was compiled using Mean Squared Error (MSE) as the loss function and the Adam optimizer for efficient gradient descent during training.

- **```python**
- **# compile the model**
- **model.compile(loss=’mean_squared_error’, optimizer=’adam’)**
- **```**

### Step 5

Training the model.

The prepared data was split into training and testing sets to assess the model’s performance. The model was then trained using the training data.

- **```python**
- **# Split data into training and testing sets**
- **X_train, X_test, y_train, y_test = train_test_split(X_scaled, y_scaled, test_size = 0.2, random_state = 42)**
- **# Train the model**
- **model.fit(X_train, y_train, epochs = 100, batch_size = 32, validation_data=(X_test, y_test))**
- **```**

### Step 6

Evaluation and validation.

The model’s performance was evaluated using various metrics, ensuring its accuracy and reliability in predicting the output parameters based on the given input parameters.

**Fig. 2:**
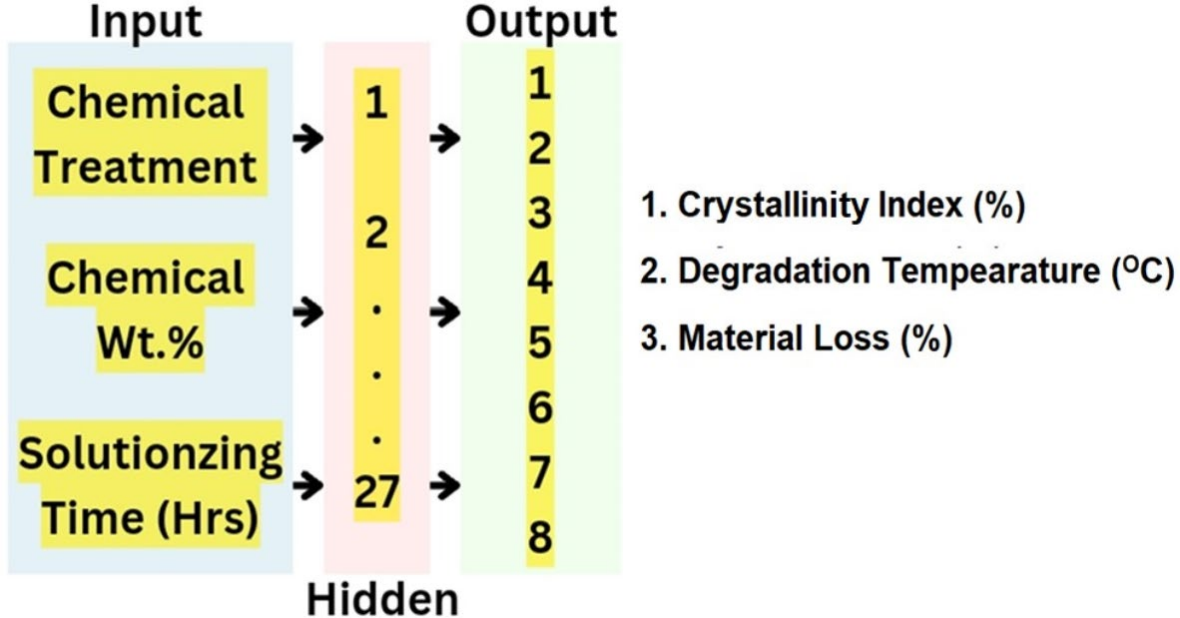
Artificial Neural Network model.

Figure 2 presents the Artificial Neural Network model.

## Results and discussions

Physical characteristics assessment on process output parameters such as crystallinity index, degradation temperature and percentage material loss after chemical treatment of HRS fibers using L_27_ orthogonal array has been discussed.

### XRD analysis and crystallinity index

**Fig. 3:**
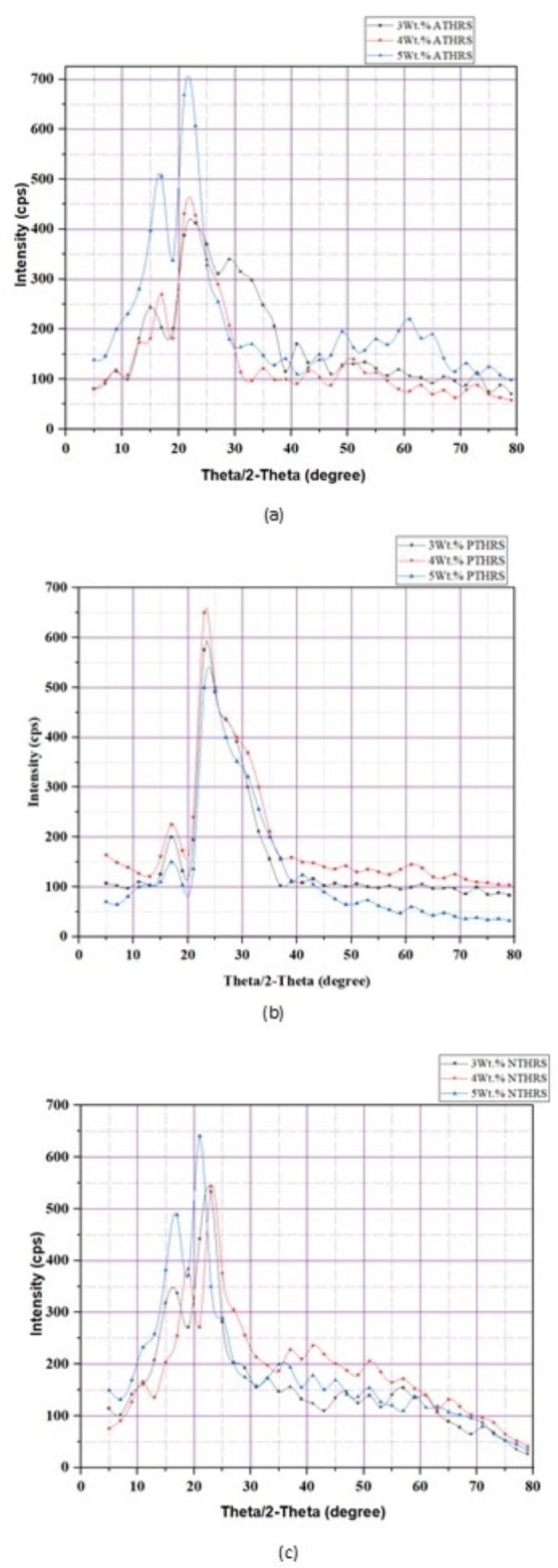
XRD analysis of HRS fibers chemically treated with; (**a**) Potassium Permanganate (KMnO_4_); (**b**) Sodium Hydroxide (NaOH); (**c**) Acetic Acid (CH_3_COOH) under various weight percentages.

 Figure 3a,b and c illustrate the intensity variations observed in the X-ray diffraction (XRD) patterns of chemically treated fibers. Two distinct peaks were identified in these graphs, which were then employed to calculate the crystallinity index of the treated fibers. The first peak (15°-20°) 2Θ, corresponds to the amorphous content of the fibers. In natural fibers, hemicellulose, lignin and the amorphous regions of cellulose contribute to this broad peak. The reduction in intensity in this region after chemical treatment indicates a decrease in the amorphous content, primarily due to the removal of hemicellulose and lignin. The broad nature of this peak is characteristic of non-crystalline structures, suggesting that the chemical treatments effectively eliminate or reduce these less-ordered regions, leading to an overall enhancement in fiber crystallinity. Further, the second peak (20°-30°) 2Θ region is associated with crystalline cellulose content in the fibers, particularly the diffraction from the (200) plane of cellulose I. The sharp peak observed in this region is indicative of the crystalline structure of cellulose. The increased intensity of this peak reflects an increase in the crystallinity content of the fibers after chemical treatment. Chemical treatments such as ATHRS, PTHRS and NTHRS enhance the crystalline structure by removing amorphous components and impurities allowing the crystalline regions of cellulose to become more prominent. The sharpness and intensity of this peak signifies that the treatments have successfully increased the degree of crystallinity within the fibers.

The percentage content of cellulose, hemicellulose, lignin, waxes, fats and pectin are presented in Table 2. Cellulose is measured by ADF (acid detergent fiber) method involving the treatment of fibers with acid detergent solution to remove hemicellulose and lignin leaving cellulose residue. The weight of the residue is measured with respect to initial fiber weight to calculate cellulose content. Further, NDF (neutral detergent fiber) method is used to remove non fibrous materials. The difference in weight between the NDF residue and ADF residue is expressed as percentage of hemicellulose. Finally, ADL test (acid detergent lignin) is used where the fibers are treated with sulfuric acid after DF treatment to dissolve cellulose, leaving Lignin as residue. Waxes, Fats and Pectin are extracted using Soxhlet extraction with organic solvents like ethanol and acetone and measured in percentage.

**Table 2:** Chemical composition of HRS fibers.

Component	Initial Composition	NaOH (4Wt.%, 3 h)	KmNO_4_ (4Wt.%, 3 h)	CH_3_COOH (4Wt.%, 3 h)
Cellulose	60%	74%	70%	65%
Hemicellulose	21%	6%	8%	11%
Lignin	14%	8%	6%	10%
Waxes, Fats and Pectin	5%	2%	2%	4%

**Fig. 4:**
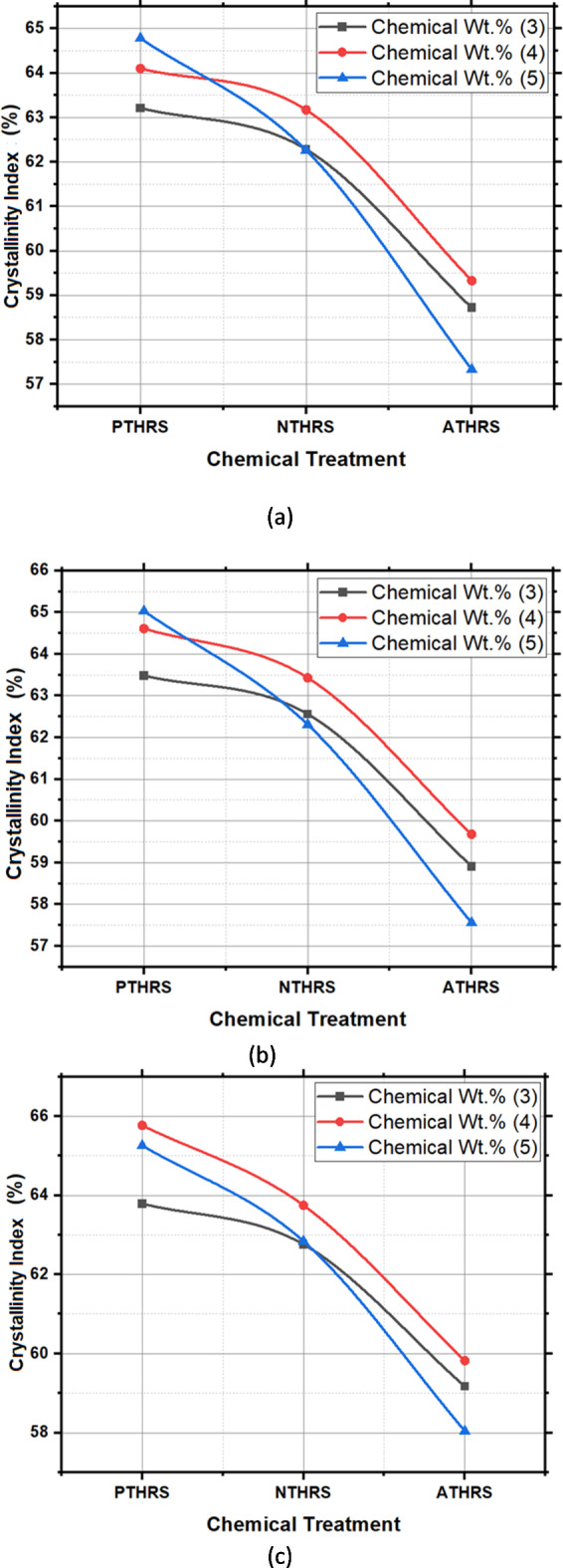
Crystallinity Index (%) of HRS fibers chemically treated with; (**a**) Solutionizing time 1 h; (**b**) Solutionizing time 2 h; (**c**) Solutionizing time 3 h.

In this study, the results highlight a compelling correlation between the choice of chemical treatment, its concentration, and solutionizing time with the resulting CI values. Potassium permanganate (PTHRS) treatment exhibited the highest CI values, reaching up to 65.77% for a chemical weight% of 4 and a solutionizing time of 3 h. This enhancement in CI can be attributed to the potent oxidizing nature of potassium permanganate, which effectively breaks down lignin, a key component in plant fibers. The oxidative degradation of lignin results in the removal of amorphous regions within the fibers, leading to a higher degree of crystallinity. Conversely, sodium hydroxide (NTHRS) and acetic acid (ATHRS) treatments, while showing notable improvements in CI, demonstrated comparatively lower values than potassium permanganate. Sodium hydroxide, being a strong alkali, initiates de-acetylation, leading to increased hydrophilicity but a slightly lower CI compared to potassium permanganate. Acetic acid, acting as a catalyst, facilitates controlled reactions, resulting in a moderate enhancement of CI. The variations in CI values among different treatments can be attributed to the specific interactions between the chemicals and the fiber components, ultimately influencing the arrangement of cellulose molecules and crystallite size. Figure 3 presents the XRD analysis of HRS fibers chemically treated with; (a) Potassium Permanganate (KMnO_4_); (b) Sodium Hydroxide (NaOH); (c) Acetic Acid (CH_3_COOH) under various weight percentages. Figure 4 presents the experimental values of the Crystallinity Index.

**Fig. 5:**
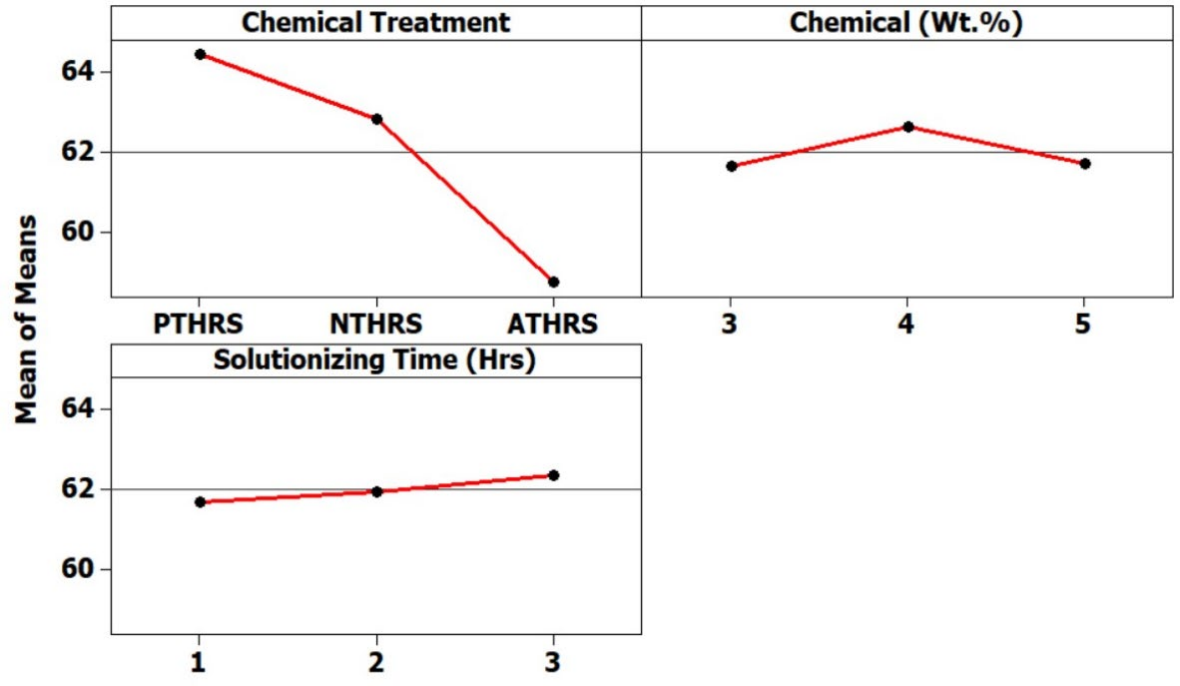
Main effects plot for Crystallinity Index of chemically treated HRS fibers.

The main effects plot presented in Fig. 5 provides a clear visual representation of the significant impact of the chemicals employed in the treatment process on the Crystallinity Index of HRS Fibers. This plot serves as a comprehensive overview, highlighting that these specific chemicals exert the most substantial influence on the Crystallinity structure of the fibers. From the main effects plot it was observed that PTHRS (Potassium Permanganate treated HRS Fibers), 4Wt.% and 3 h solutionizing time are the most influencing parameters to achieve highest Crystallinity Index value (65.58%). Further, ATHRS has found to have least effect on the Crystallinity Index of the HRS fibers (57.64%).

**Fig. 6:**
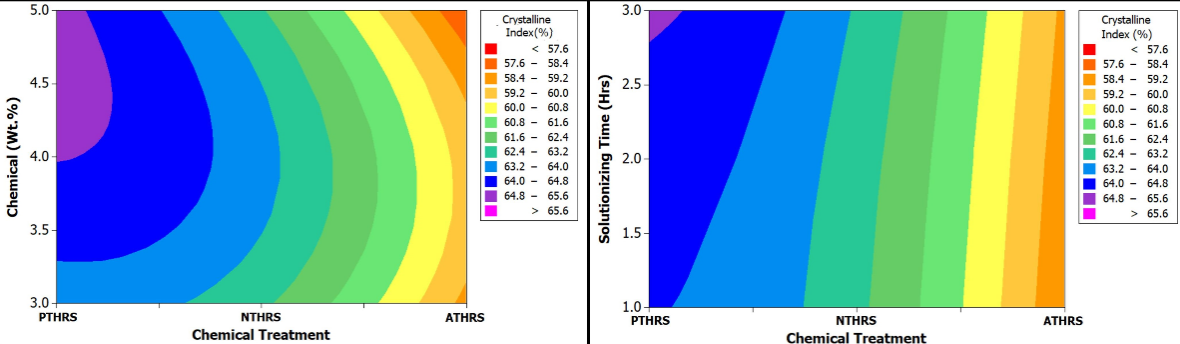
Spectrum plots for Crystallinity Index of chemically treated HRS fibers.

Figure 6 offers a more detailed insight by displaying a spectrum plot. This plot meticulously maps out the variation in Crystallinity Index values across different weight percentages and solutionizing time intervals of HRS fibers subjected to diverse chemical treatments. It effectively captures the nuanced relationship between these variables, shedding light on the intricate dynamics of crystallinity under varying experimental conditions. From Fig. 6 it was observed that PTHRS chemical treatment with increased chemical weight% and increased solutionizing time there was considerable increase in Crystallinity Index of more than 65.6%.

**Fig. 7:**
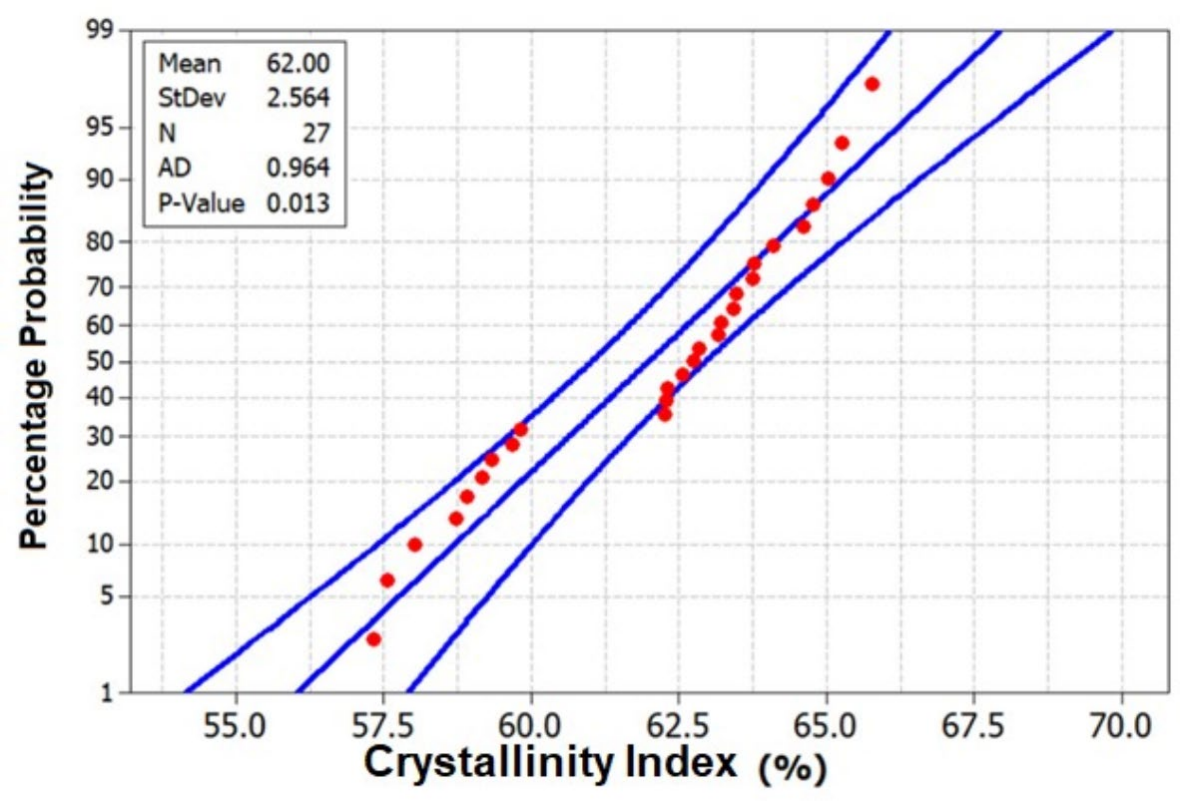
Probability plot for Crystallinity Index of chemically treated HRS fibers.

Furthermore, Fig. 7, the probability plot for Crystallinity Index, provides a statistical perspective, offering a probabilistic representation of the data distribution. This plot not only reinforces the trends observed in Figs. 5 and 6 but also quantifies the likelihood of specific Crystallinity Index values occurring within the dataset, adding a layer of probabilistic understanding to the analysis. Hence from the probability plot it was observed that there was a drastic increase from 57.5 to 65.6% for chemical treatment (PTHRS, NTHRS and ATHRS) of HRS Fibers. However, PTHRS shows the highest probability of getting maximum Crystallinity Index of 65.6%.

**Fig. 8:**
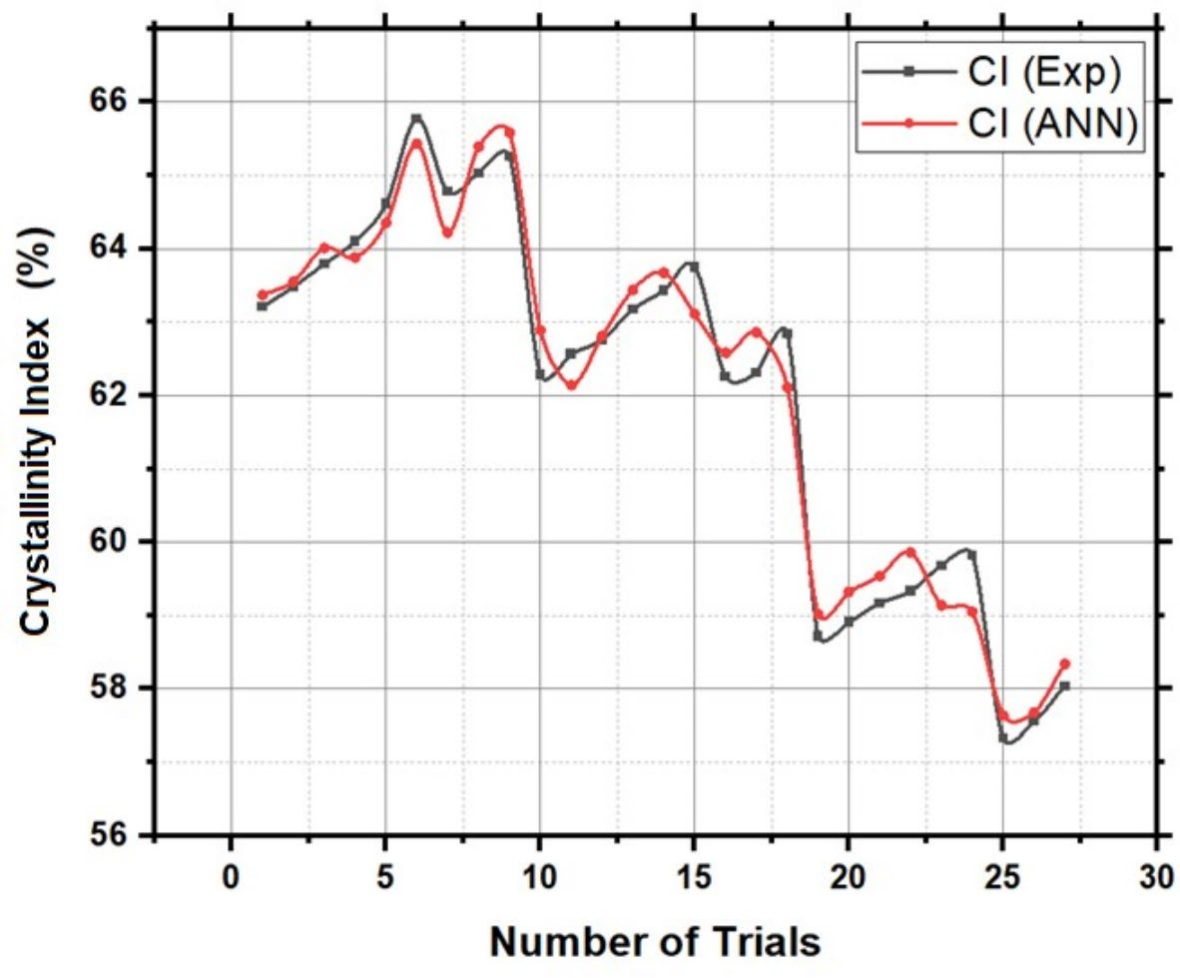
Validation of ANN derived Crystallinity Index values using experimental values.

Figure 8 provides validation of ANN predicted values of Crystallinity Index using experimental values. The Back-propagation Artificial Neural network was successfully applied to predict the values of Crystallinity Index with an accuracy of 98.66%. Table 3 presents the performance metrics of the Artificial Neural Network for Crystallinity Index.

**Table 3:** Performance Metrics of Artificial neural network.

Metric	Value
Learning Rate	0.01
Precision (%)	93.6
Recall (%)	93.2
F1-Score (%)	95.2
MAE	0.018
RMSE	0.024
R^2^	0.91
MAPE	2.73

### Thermo-gravimetric analysis

Thermo-gravimetric Analysis (TGA) is an analytical technique used to study the thermal stability and composition of materials. In TGA, a sample is subjected to a controlled temperature program and change in weight are measured as a function of temperature or time. This method is particularly valuable in understanding the thermal decomposition, degradation, and volatilization of materials. By analyzing the material loss or gain of a sample as it is heated or cooled, we can determine various properties, including purity, composition, and stability. As the temperature increases, the sample undergoes thermal decomposition, leading to a gradual reduction in weight.

The enhanced thermal stability of chemically treated Hibiscus Rosa-Sinensis fibers is primarily due to the combined effects of removing hemicellulose and extractives, which are more thermally labile than cellulose and lignin, and increasing the crystallinity of cellulose. The treatments effectively eliminate these thermally unstable components and enhance the crystalline structure of cellulose, thereby shifting the onset of degradation to higher temperatures. Additionally, the cleaning of the fiber surface from impurities further contributes to improved thermal behaviour. Therefore, the outstanding thermal stability is not solely due to the elimination of lignin but results from the comprehensive modification of the composition and structure of HRS Fiber.

**Fig. 9:**
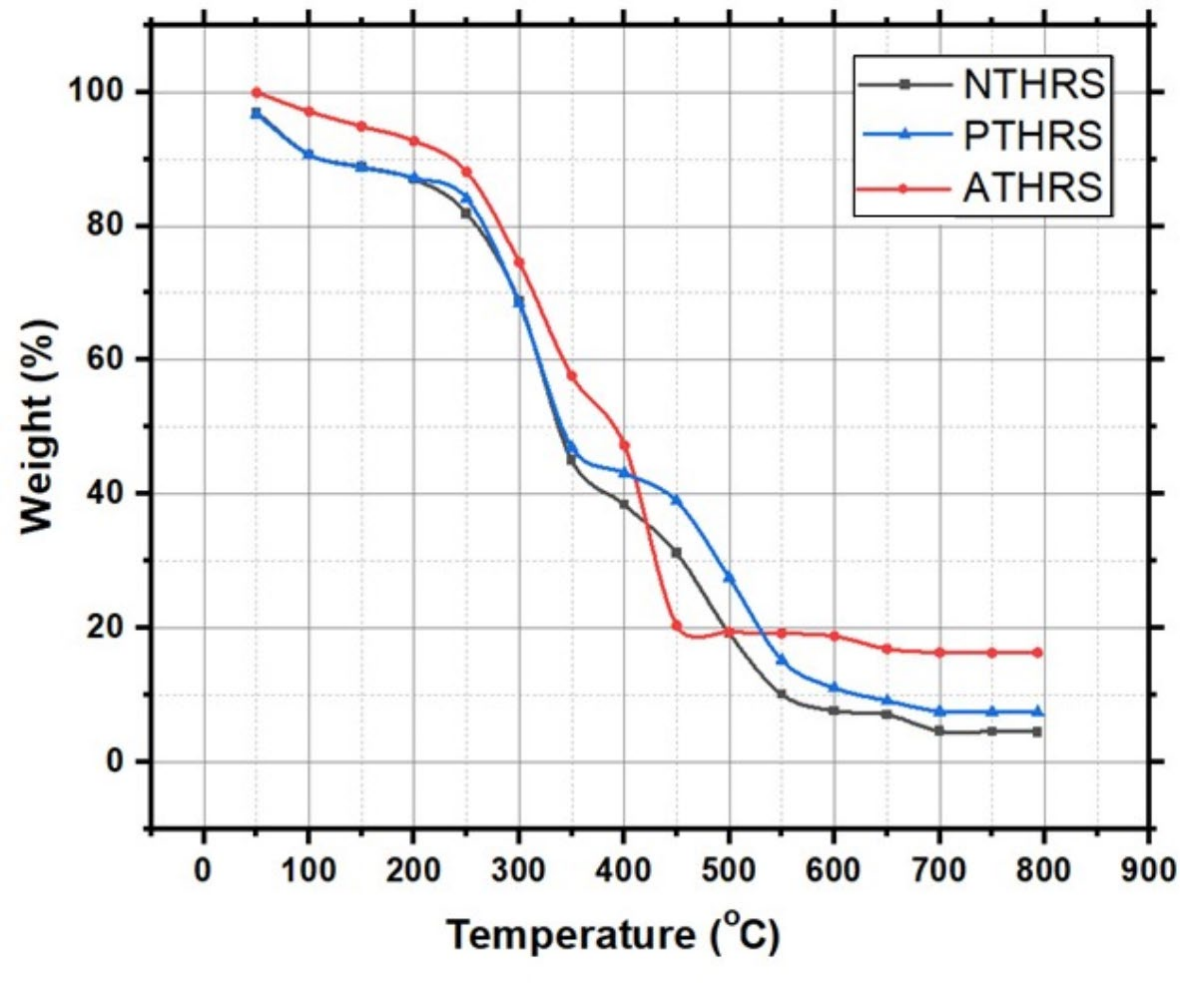
Percentage Weight loss post chemical treatment of fibers with 4Wt.%.

The graph (Fig. 9) showcases the thermally induced changes in the material, providing valuable insights into its thermal behavior. These insights are essential in numerous fields, including chemistry, materials science, and environmental science, where understanding how materials respond to temperature variations is crucial for designing efficient and stable products and processes.

### Degradation temperature

**Fig. 10:**
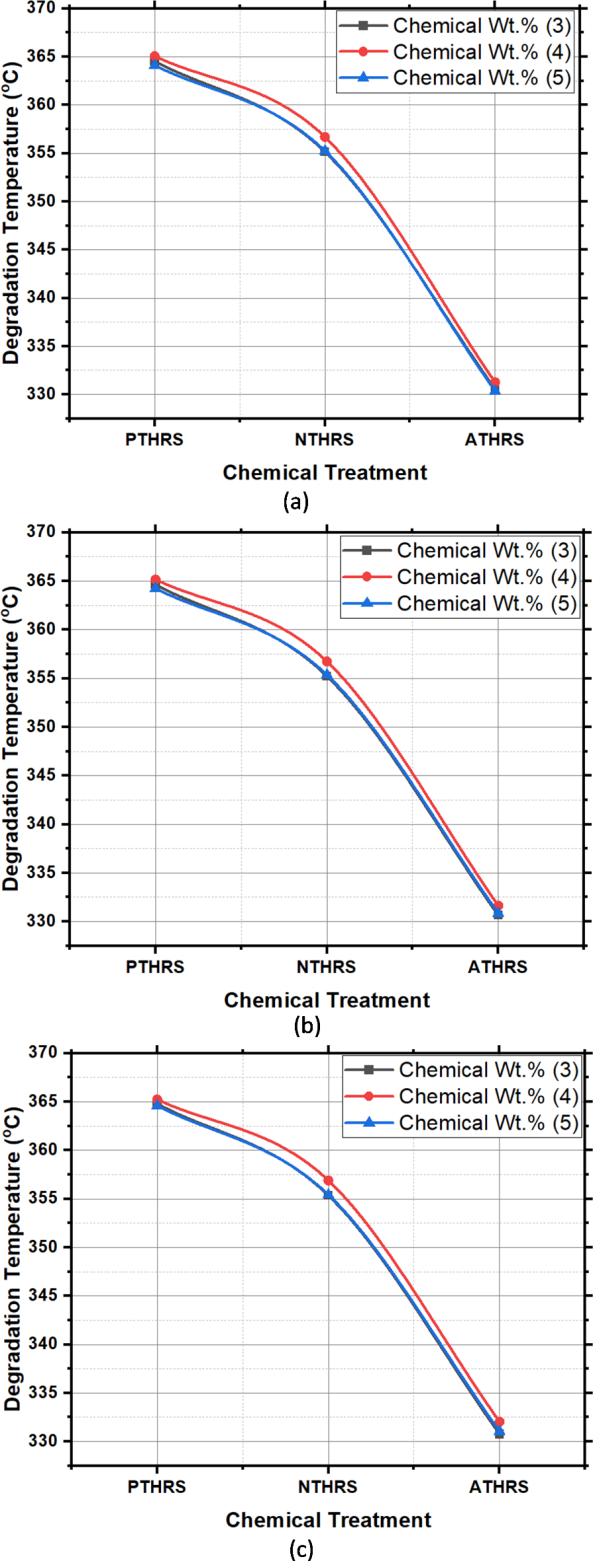
Degradation Temperature (^o^C) of HRS fibers chemically treated with; (**a**) Solutionizing time 1 h; (**b**) Solutionizing time 2 h; (**c**) Solutionizing time 3 h.

Degradation temperature, a key indicator of the fiber thermal stability, exhibited compelling variations across different treatments (Fig. 10). Potassium permanganate (PTHRS) treatment at 4 Wt.% and 3 h of solutionizing time resulted in a notably high degradation temperature of 365.24 °C. This exceptional thermal stability can be attributed to the efficient removal of impurities, particularly lignin, which is susceptible to thermal degradation. The preservation of essential fiber components was achieved through the selective action of potassium permanganate, ensuring the structural integrity of HRS fibers even under high-temperature conditions. As presented in Fig. 10 Sodium hydroxide treatment at 3 Wt.% and 3 h exhibited a degradation temperature of 355.35 °C and Acetic acid treatment at 4 Wt.% and 3 h exhibited a degradation temperature of 332.03 °C. The chemically treated Hibiscus fibers can withstand high degradation temperature of compared to other natural fibers^31^.

**Fig. 11:**
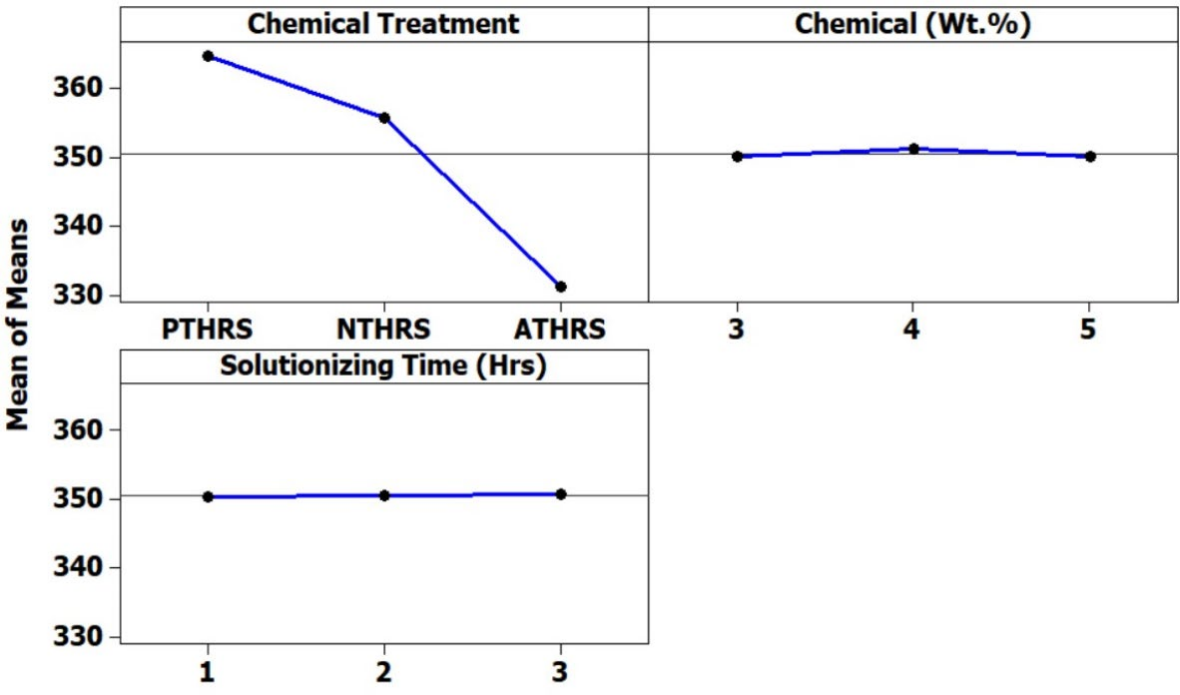
Main effects plot for Degradation Temperature of chemically treated HRS fibers.

**Fig. 12:**
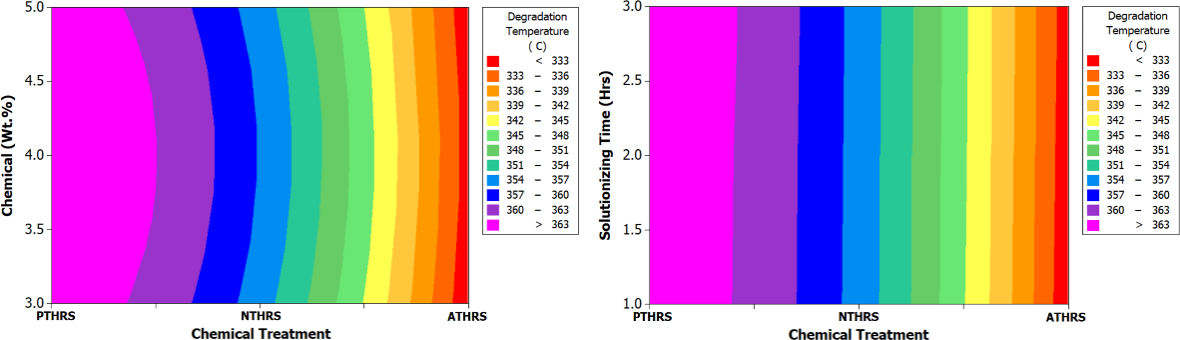
Spectrum plots for Degradation Temperature of chemically treated HRS fibers.

In Fig. 11, the main effects plot emphatically highlights the significant influence of treatment chemicals on the degradation temperature of HRS Fibers. This observation is crucial, as degradation temperature serves as a key indicator of the thermal stability and overall robustness of materials under various conditions. A higher degradation temperature often signifies enhanced resistance to thermal stress, making it a critical parameter in applications where temperature fluctuations are common. Figure 12, the spectrum plot, meticulously delineates the variations in degradation temperature values across different weight percentages and solutionizing timeframes in diverse chemical environments. This detailed analysis provides valuable insights into how different treatments affect the fibers’ thermal stability, offering essential information for material engineering and processing.

**Fig. 13:**
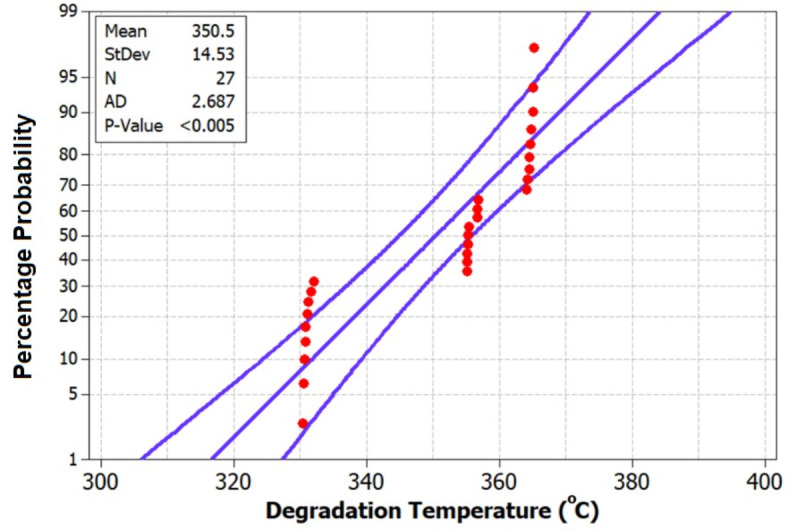
Probability plot for Degradation Temperature of chemically treated HRS fibers.

**Fig. 14:**
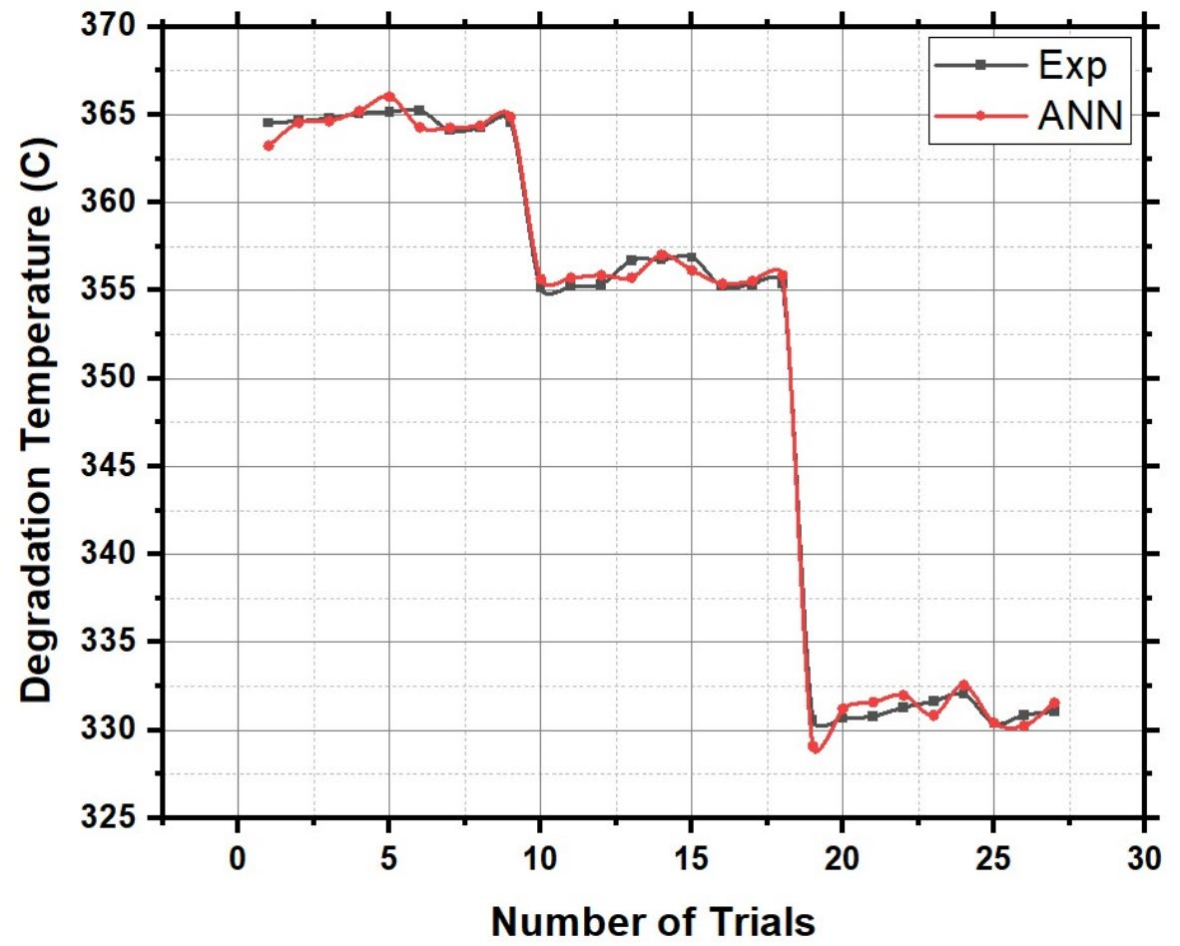
Validation of ANN predicted Degradation Temperature using experimental results.

Figure 13, the probability plot, offers a statistical perspective by illustrating the distribution of degradation temperature values within the dataset. This probabilistic view enhances the analysis, providing a comprehensive understanding of the variability in the thermal stability of HRS Fibers under different treatment conditions. Furthermore, Fig. 14, the validation plot, plays a pivotal role in confirming the accuracy of the Artificial Neural Network (ANN) predictions for degradation temperature. This validation step ensures the reliability and applicability of the predictive model, allowing for precise estimation of degradation temperatures based on the treatment parameters.The Back-propagation Artificial Neural network was successfully applied to predict the values of Degradation Temperature with an accuracy of 94.77%. Table 4 presents the performance metrics of the Artificial Neural Network for Degradation Temperature.

**Table 4:** Performance Metrics of Artificial neural network.

Metric	Value
Learning Rate	0.01
Precision (%)	94.5
Recall (%)	94.7
F1-Score (%)	92.4
MAE	0.029
RMSE	0.036
R^2^	0.87
MAPE	2.69

### Material loss

**Fig. 15:**
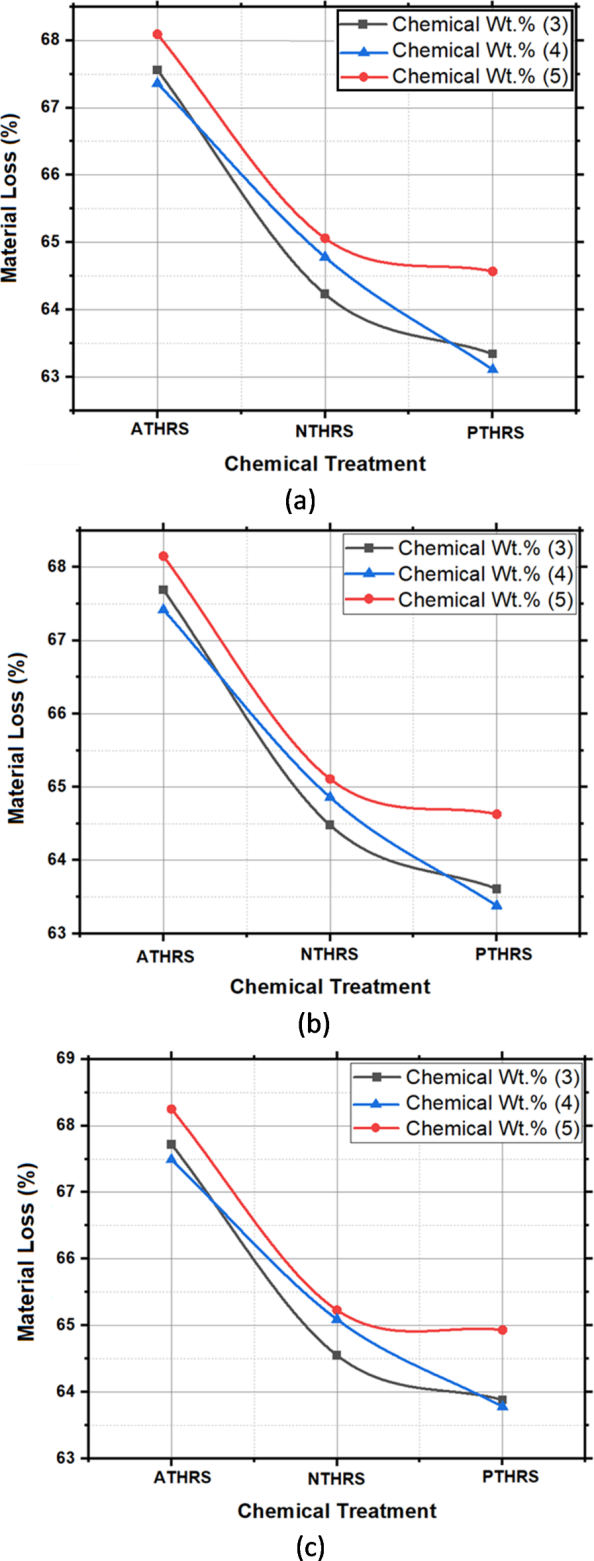
Material Loss (%) of HRS fibers chemically treated with; (**a**) Solutionizing time 1 h; (**b**) Solutionizing time 2 h; (**c**) Solutionizing time 3 h.

Percentage Material loss, representing the extent of impurity removal and fiber purity, also showcased intriguing patterns. Potassium permanganate treatment at 4 Wt.% and 3 h exhibited a low Material loss of 63.11%, indicating the successful elimination of impurities and non-essential components. This result aligns with the high degradation temperature observed, emphasizing the correlation between impurity removal and enhanced thermal stability. Sodium hydroxide (NTHRS) and acetic acid (ATHRS) treatments, while displaying slightly lower degradation temperatures and higher percentage material losses than potassium permanganate, still demonstrated substantial improvements compared to untreated fibers. As presented in Fig. 15, thematerial loss of 64.55%, highlighting the efficacy of alkali treatment in enhancing fiber purity and thermal stability. Acetic acid treatment at 4 Wt.% and 3 h exhibited a material loss of 68.25%, emphasizing the controlled reactions facilitated by acetic acid, ensuring the removal of impurities while preserving the essential components of the fibers.

**Fig. 16:**
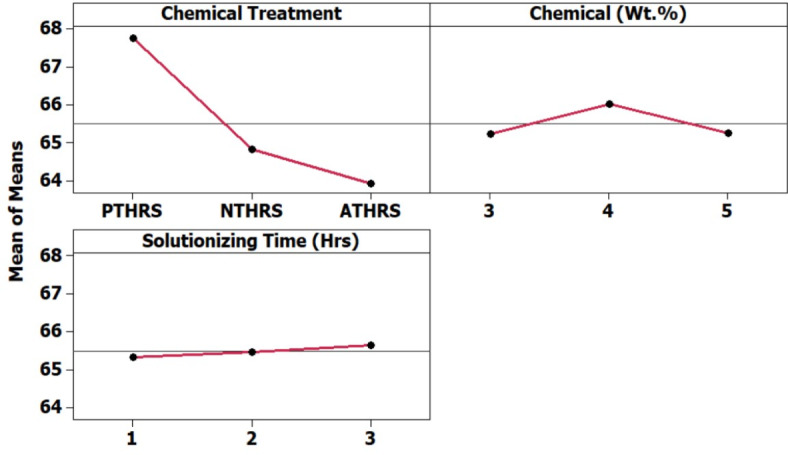
Main effects plot for Material Loss of chemically treated HRS fibers.

**Fig. 17:**
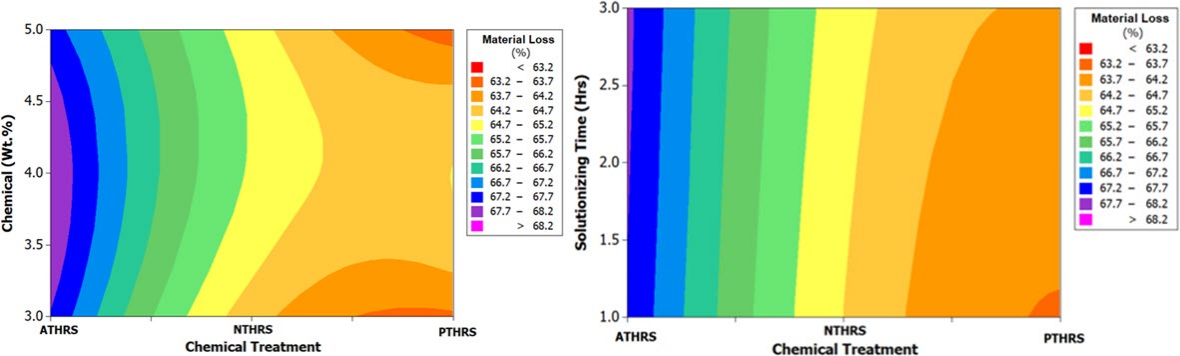
Spectrum plots for Material Loss of chemically treated HRS fibers.

In Fig. 16, the main effects plot prominently demonstrates the significant impact of treatment chemicals on the material loss of HRS Fibers. This parameter is pivotal for understanding the material’s stability and its response to various treatment conditions, providing essential insights for industrial and research applications. Figure 17, the spectrum plot, meticulously delineates the variations in material loss values across different weight percentages and solutionizing timeframes in diverse chemical environments. This detailed analysis offers a nuanced understanding of how different treatments influence the fibers’ mass loss, shedding light on their stability under varied experimental conditions.

**Fig. 18:**
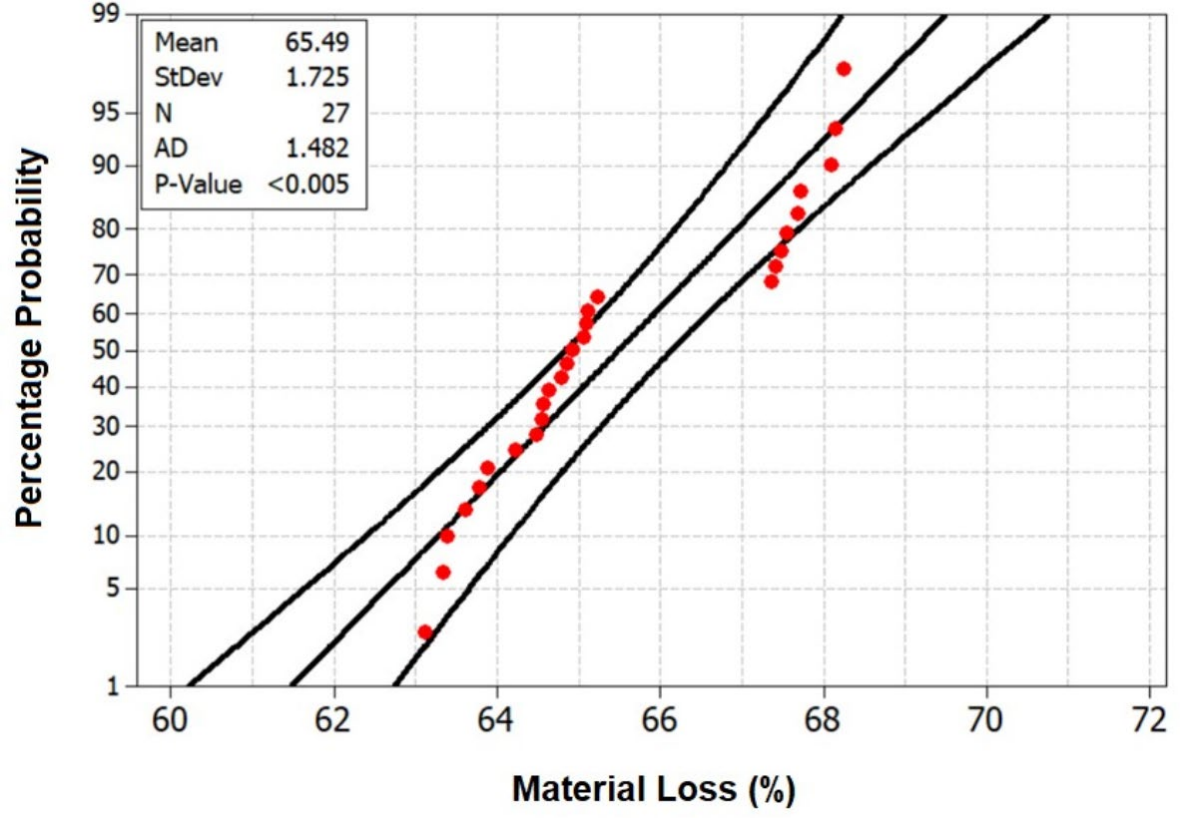
Probability plot for Material Loss of chemically treated HRS fibers.

**Fig. 19:**
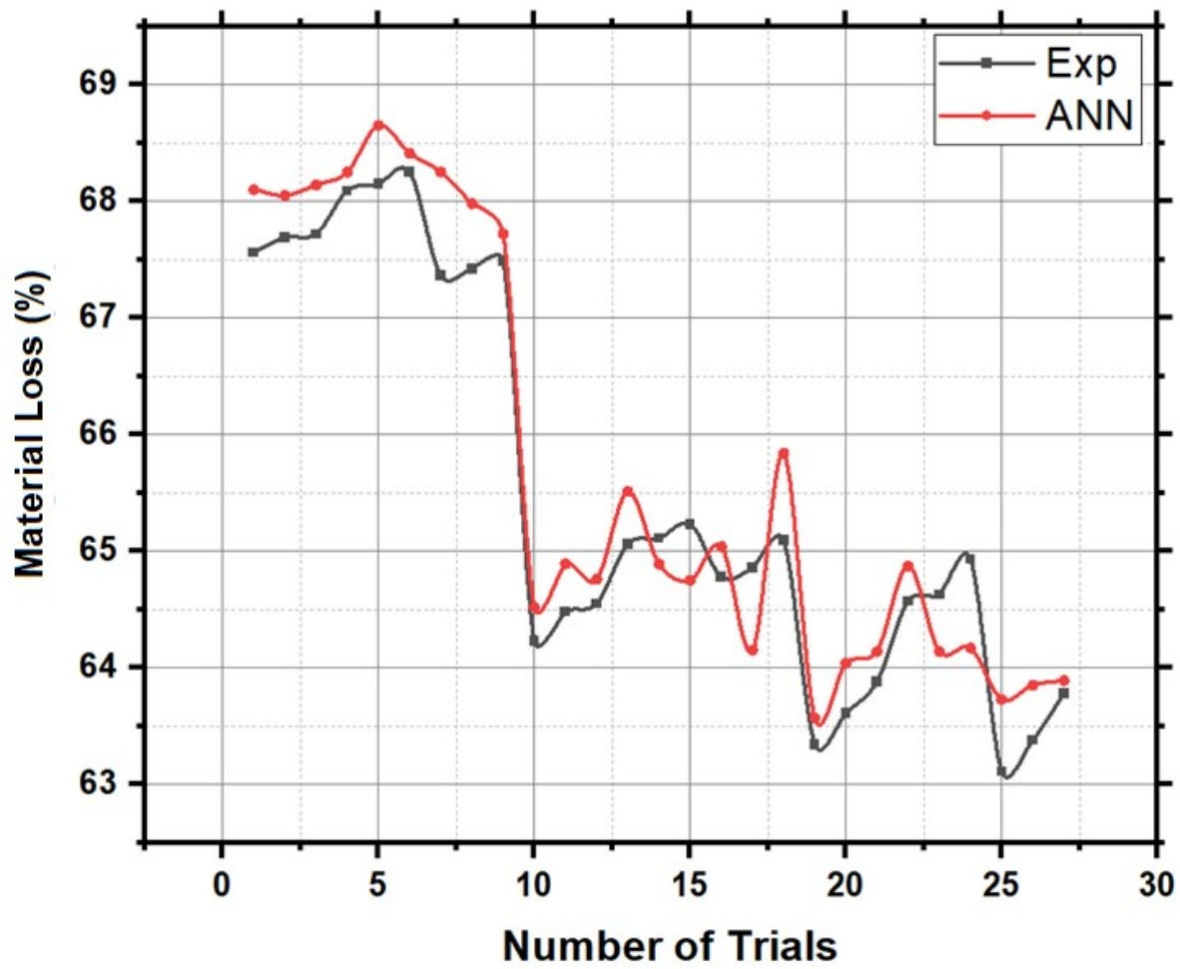
Validation of ANN predicted Material Loss using experimental results.

Figure 18, the probability plot, provides a statistical perspective by illustrating the distribution of material loss values within the dataset. This probabilistic view enhances the analysis, offering a comprehensive understanding of the variability in material loss under different treatment scenarios. Furthermore, Fig. 19, the validation plot, plays a pivotal role in confirming the accuracy of the Artificial Neural Network (ANN) predictions for material loss. This validation step ensures the reliability and applicability of the predictive model, allowing for precise estimation of material loss based on the treatment parameters. Together, these analyses provide a comprehensive and reliable understanding of how treatment chemicals significantly influence the material loss of HRS Fibers, essential information for industries and researchers involved in material development and optimization. The Back-propagation Artificial Neural network was successfully applied to predict the values of Material Loss with an accuracy of 95.44%. Table 5 presents the performance metrics of the Artificial Neural Network for Material Loss.

**Table 5:** Performance Metrics of Artificial neural network.

Metric	Value
Learning Rate	0.01
Precision (%)	91.2
Recall (%)	91.7
F1-Score (%)	93.4
MAE	0.032
RMSE	0.037
R^2^	0.89
MAPE	3.46

### Microscopic analysis of chemically treated HRS fibers

**Fig. 20:**
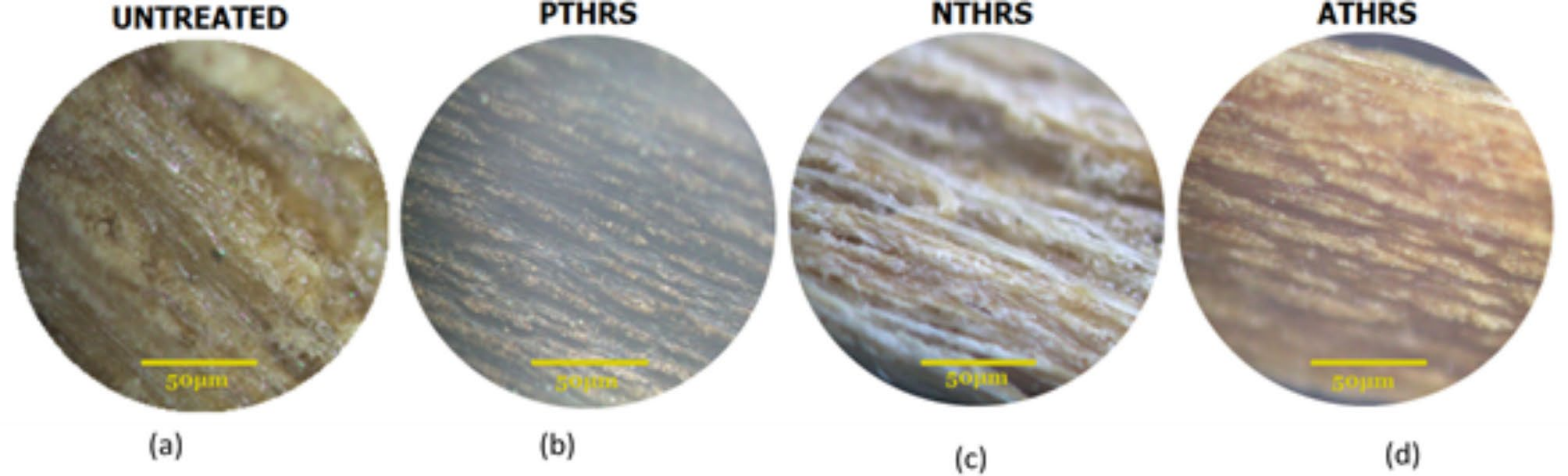
Optical micrograph of HRS fibers (**a**) Untreated (**b**) Potassium Permanganate (PTHRS) (**c**) Sodium Hydroxide (NTHRS) (**d**) Acetic Acid (ATHRS).

Figure 20 (a-d) provides a comprehensive visual comparison of the microstructures of HRS Fibers under different treatments. The optical microscope images reveal distinct differences among the untreated fibers, fibers treated with Potassium Permanganate, Sodium Hydroxide, and Acetic Acid. Remarkably, the Potassium Permanganate treatment (Fig. 20b) exhibits the most favorable results, displaying a well-defined and uniform microstructure. This suggests a successful alteration at the microscopic level, potentially indicating enhanced fiber integrity and reduced defects. In comparison, the Sodium Hydroxide treatment (Fig. 20c) shows moderate improvements, with a relatively smoother microstructure compared to the untreated fibers (Fig. 20a). Finally, the Acetic Acid treatment (Fig. 20d) appears to have the least impact, as the microstructure still displays notable irregularities and imperfections. This microstructural analysis aligns with the treatment effectiveness, indicating that Potassium Permanganate treatment has the most significant positive impact, followed by Sodium Hydroxide, while Acetic Acid treatment shows comparatively limited improvements. These findings provide valuable insights into the effectiveness of different chemical treatments on the microstructural integrity of HRS Fibers, guiding further research and development efforts in material science and engineering.

**Fig. 21:**
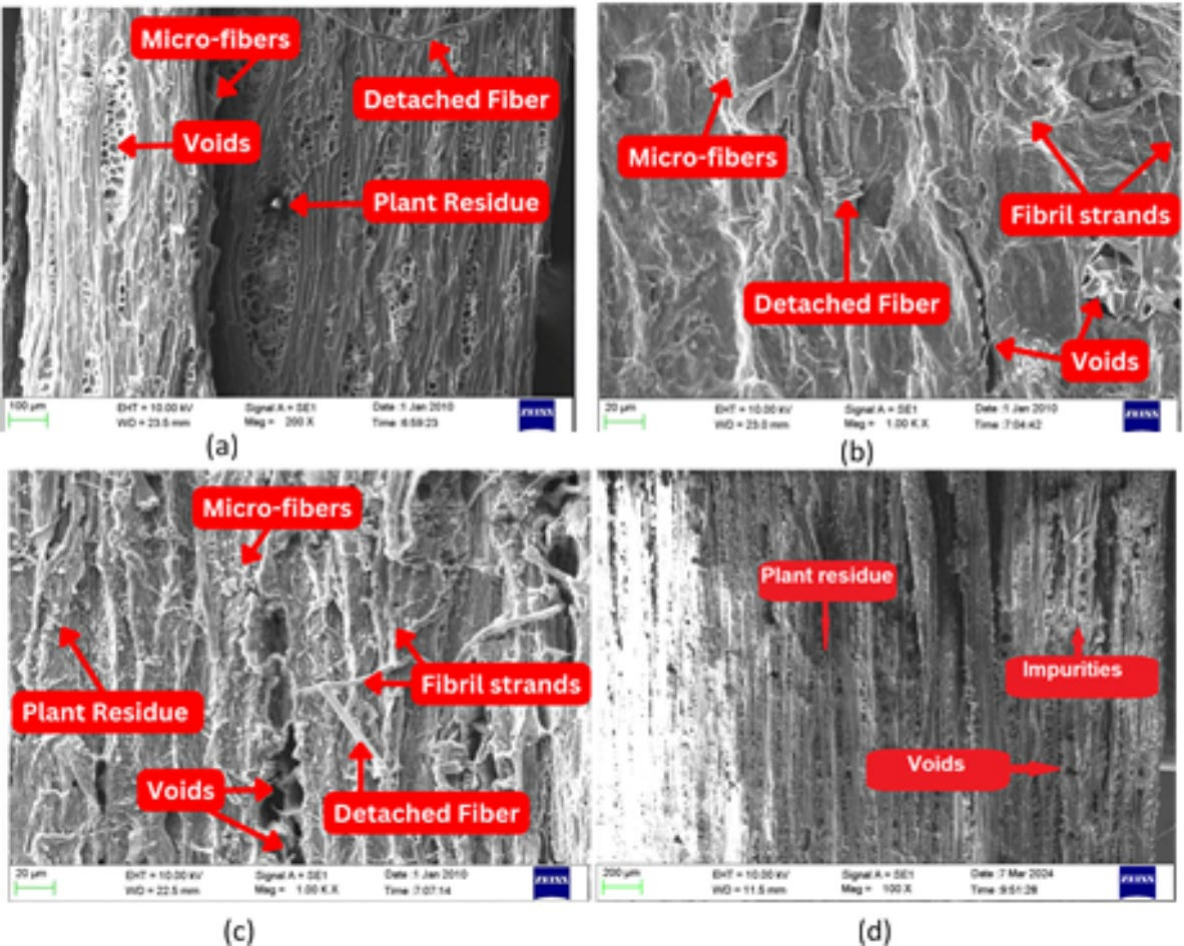
Scanning electron microscope images of HRS fibers treated with; (**a**) Sodium Hydroxide (NTHRS); (**b**) Potassium Permanganate (PTHRS) (**c**) Acetic Acid (ATHRS); (**d**) Untreated fiber.

In this study, Hibiscus Rosa-Sinensis (HRS) plant fibers were subjected to different chemical treatments, namely NaOH (NTHRS), KMnO_4_ (PTHRS), and CH_3_COOH (ATHRS). Scanning Electron Microscopy (SEM) analysis was performed to analyze the structure and topography of these treated fibers. The SEM Images of NTHRS fibers (Fig. 21a) revealed that the fibers consisted of microfibers closely attached by pectin. The diameter of NTHRS fibers ranged from 199.9 μm to 314.8 μm, with fibrils ranging from 3.16 μm to 18.3 μm. However, undesired organic attachments were observed on the fiber surface, leading to poor adhesion with the matrix phase. Further, Potassium Permanganate treatment effectively removed hemicellulose, lignin, wax, and pectin, resulting in a clean and rough fiber surface. PTHRS fibers (Fig. 21b) exhibited significantly reduced impurities, leading to a cleaner surface compared to NTHRS. The diameter of PTHRS fibers ranged from 145.6 μm to 286.1 μm, and fibrils ranged from 2.84 μm to 16.46 μm. The treatment enhanced cellulose structures and surface roughness, improving adhesion capability with the matrix. In contrast, ATHRS fibers (Fig. 21c) displayed higher surface roughness and voids due to the removal of pectin/lignin. The fibrils in ATHRS appeared slightly detached, impacting the overall adhesion properties. Treatment with CH_3_COOH resulted in a rougher surface texture and reduced fiber and fibril diameters. These observations suggest that chemical treatments play a crucial role in modifying the surface properties of HRS fibers. NaOH treatment, while effective in removing impurities, led to undesired organic attachments, affecting adhesion. KMnO_4_ treatment improved cellulose structures and reduced impurities, enhancing adhesion capability. CH_3_COOH treatment, although increasing surface roughness, caused fibril detachment, potentially impacting the mechanical properties of the fibers. High weight% (9 wt%) of sodium hydroxide (NaOH) treatment improved the surface texture of the fiber^32^. The Fig. 21 d represents the SEM image of untreated fiber. The impurities like wax and lignin were present in the surface of the fiber.

**Fig. 22:**
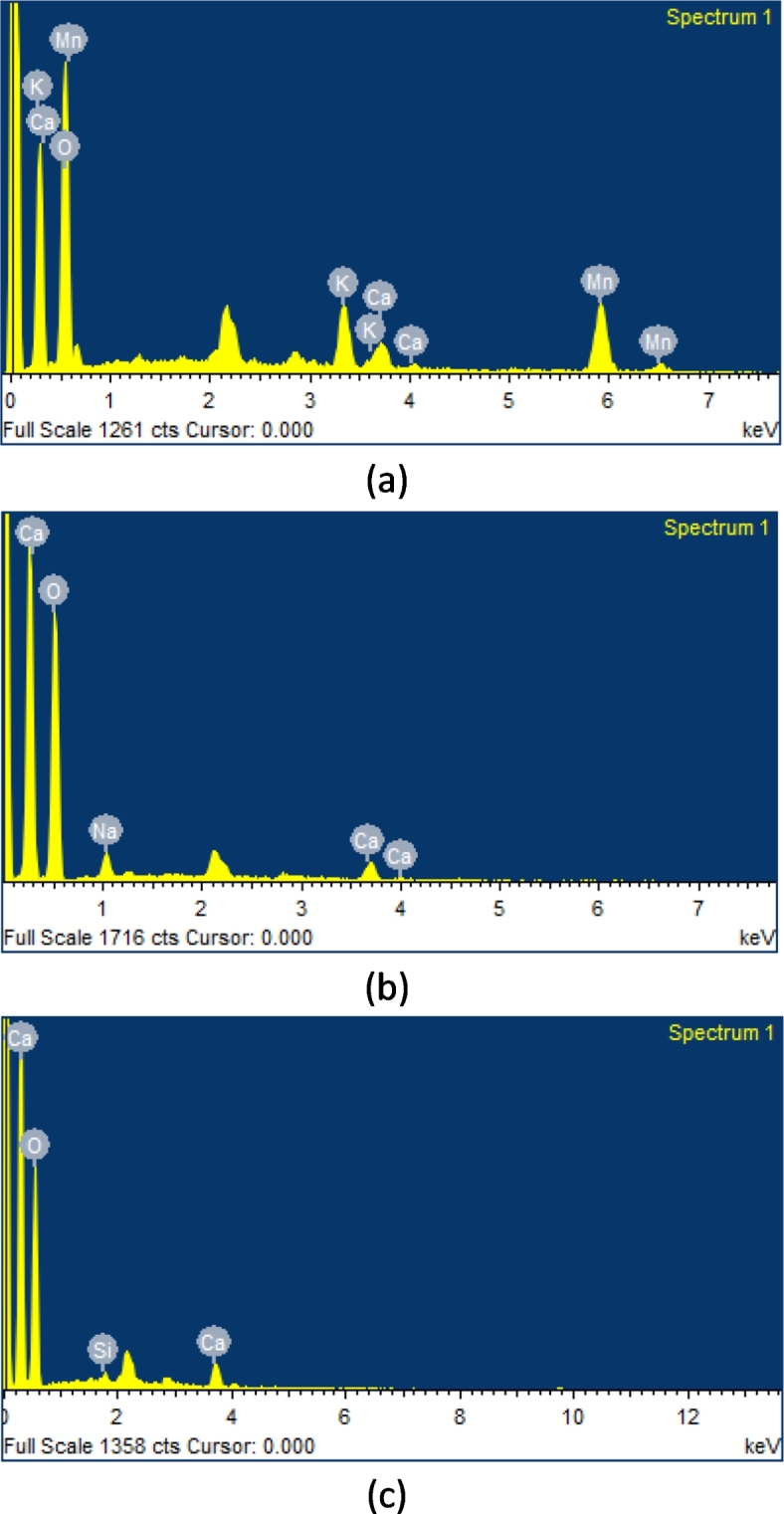
EDS (elemental distribution) images of HRS fibers treated with; (**a**) Potassium Permanganate (PTHRS); (**b**) Sodium Hydroxide (NTHRS); (**c**) Acetic Acid (ATHRS).

In this comprehensive study, Hibiscus Rosa-Sinensis (HRS) plant fibers underwent treatment with three distinct chemical agents: Potassium Permanganate (PTHRS), Sodium Hydroxide (NTHRS), and Acetic Acid (ATHRS). Through meticulous Energy Dispersive X-ray Spectroscopy (EDS) analyses, the elemental compositions of these treated fibers were determined (Table 2), giving insights into their chemical transformations. In the case of PTHRS fibers (Fig. 22a), the high oxygen content indicated the presence of cellulose and hemicellulose, essential organic components of plant fibers, alongside the incorporation of potassium, calcium, and manganese. This unique elemental profile suggests the successful interaction of the fibers with potassium permanganate, potentially enhancing their mechanical and chemical properties for specialized applications, such as in reinforced composite materials. Further, within NTHRS fibers (Fig. 22b), the dominant oxygen presence proves the retention of cellulose and hemicellulose structures. The detection of sodium and minor calcium content suggested the interaction of the fibers with sodium hydroxide, leading to specific chemical modifications. Sodium hydroxide treatment is known to remove impurities and enhance fiber surface properties, making the fibers suitable for applications requiring improved adhesion and compatibility with other materials. Finally, ATHRS fibers (Fig. 22c) exhibited elevated oxygen levels, indicative of cellulose content, along with the presence of sodium and calcium. Acetic acid treatment is often employed to eliminate undesired attachments, creating a rougher surface texture ideal for enhanced adhesion in composite materials. The retained sodium and calcium elements suggest the preservation of certain plant components, highlighting the complexity of chemical interactions during the treatment process. Table 6 presents the percentage elemental distribution in HRS fibers treated with different chemicals.

**Table 6:** Percentage elemental distribution in HRS fibers treated with different chemicals.

Chemical Treatment	Element	Weight%	Atomic %
ATHRS	O (K)	88.23	94.65
	Si (K)	1.70	1.04
	Ca (K)	10.07	4.31
	Total	100.00	
NTHRS	O (K)	86.33	92.12
	Na (K)	6.49	4.82
	Ca (K)	7.18	3.06
	Total	100.00	
PTHRS	O (K)	54.12	78.42
	K (K)	9.01	5.34
	Ca (K)	4.34	2.51
	Mn (K)	32.53	13.73
	Total	100.00	

## Conclusions

The research investigates the effect of chemical treatments—Potassium Permanganate (KMnO₄), Sodium Hydroxide (NaOH), and Acetic Acid (CH₃COOH)—on the Crystallinity Index (CI), thermal stability, material loss, and microstructural properties of Hibiscus Rosa-Sinensis (HRS) plant fibers. Potassium Permanganate treatment, due to its potent oxidizing nature, achieved the highest CI of 65.77% by effectively breaking down lignin and removing amorphous regions. Sodium Hydroxide and Acetic Acid treatments also improved CI but to a lesser extent, with NaOH enhancing hydrophilicity and CH₃COOH facilitating controlled reactions for moderate CI enhancement. In terms of thermal stability, Potassium Permanganate-treated fibers displayed a degradation temperature of 365.24 °C, outperforming NaOH (355.35 °C) and CH₃COOH (332.03 °C) treatments. Material loss was quantified, revealing low losses for KMnO₄ (63.11%) and slightly higher but still effective impurity removal for NaOH (64.55%) and CH₃COOH (64.93%). Microstructural analysis showed that NaOH effectively removed impurities but could lead to undesired organic attachments, KMnO₄ enhanced cellulose structures and reduced impurities, and CH₃COOH increased surface roughness but could cause fibril detachment. The back-propagation artificial neural network successfully predicted CI, degradation temperature, and material loss with accuracies of 98.66%, 94.77%, and 95.44%, respectively. These findings are crucial for optimizing chemical treatments to improve the structural and thermal properties of natural fibers for composite applications.

## Limitations and future scope

### Limitations of the study

This study, while thorough in its approach to enhancing and characterizing Hibiscus Rosa-Sinensis (HRS) fibers, faces several limitations. Firstly, the scope of chemical treatments is restricted to Sodium Hydroxide (NaOH), Potassium Permanganate (KMnO4), and Acetic Acid (CH3COOH). Exploring a broader range of chemical agents or combinations could provide a more comprehensive understanding of how different treatments affect fiber properties. Additionally, the study relies on a fixed set of treatment parameters defined by Taguchi’s L_27_ orthogonal array, potentially overlooking optimal conditions outside this matrix. Another limitation is the exclusive focus on the crystallinity index and thermal stability; mechanical testing of composite materials made from these fibers could offer more practical insights into performance. Furthermore, the environmental impact and safety of the chemical treatments have not been addressed, which is critical for the industrial application of these processes. Finally, the modeling with Artificial Neural Networks (ANN) assumes idealized conditions that might not fully capture the complexities of real-world scenarios, potentially limiting the applicability of the findings.

### Future scope for the study

Building on the findings of this study, future research can explore several promising avenues. Extending the range of chemical treatments to include enzymatic and biotechnological methods could offer eco-friendly alternatives for fiber modification. Investigating the effects of varying concentrations and combinations of these chemicals, as well as exploring other natural fibers, would provide broader insights into optimizing fiber properties. Future studies should also incorporate mechanical testing of composite materials made from treated fibers to validate their practical performance and durability. Additionally, life cycle assessments and environmental impact analyses of the chemical treatments should be conducted to ensure sustainable and safe industrial applications. The integration of advanced modeling techniques, such as machine learning algorithms, could further refine the predictive accuracy for fiber treatment outcomes. Lastly, examining the economic feasibility and scalability of these treatments in commercial applications would bridge the gap between laboratory research and industrial utilization.

## Data Availability

All data generated or analysed during this study are included in this published article.
